# Association Between Drinking Water Sources and Osteoarthritis Incidence in Chinese Elderly Population: National Population-Based Cohort Study

**DOI:** 10.3389/fmed.2021.759514

**Published:** 2022-02-03

**Authors:** Yangchang Zhang, Tingting Wu, Shisi Shen, Yang Xiong, Xu Wang, Jialu Yang, Xirui Chen, Yanjun Lu, Xun Lei

**Affiliations:** ^1^Department of Public Health, Chongqing Medical University, Chongqing, China; ^2^Research Center for Medicine and Social Development, Chongqing Medical University, Chongqing, China; ^3^The Innovation Center for Social Risk Governance in Health, Chongqing Medical University, Chongqing, China; ^4^Chongqing Collaborative Innovation Center for Functional Food, Chongqing University of Education, Chongqing, China; ^5^The First School of Clinical Medicine, Chongqing Medical University, Chongqing, China; ^6^Department of Urology, The West China Hospital, Sichuan University, Chengdu, China; ^7^Research Center for Public Health Security, Chongqing Medical University, Chongqing, China

**Keywords:** drinking water, well water, spring water, surface water, tap water, osteoarthritis

## Abstract

**Background:**

The elderly is a vulnerable group susceptible to toxic environmental factors; however, the association between unsafe water and osteoarthritis (OA) incident among this population is poorly investigated. This study aimed to examine the effects of drinking water sources on OA risk in the Chinese elderly population.

**Methods:**

Data were obtained from the China Longitudinal Healthy Longevity Survey to perform a 16-year longitudinal study. All participants aged ≥65 years at baseline were potentially eligible. Information on drinking water sources was recorded via a structured questionnaire. The water source was classified as well, surface, spring, or tap. OA was selected as the health outcome. Cox proportional hazard models, adjusted socio-demographics, lifestyle, and chronic disease were used to estimate the hazard ratio (HR) for OA. Subgroup analyses were performed to assess the potential interactive effect.

**Results:**

A total of 12,543 participants were included. With restriction to the longest follow-up time, the mean survival time of follow-up was 14.56 years. Among the cohort members, 1,585 members suffered from OA. In the full model, drinking from well water and spring water was associated with a higher risk of OA compared with drinking from tap water (HR: 1.37, 95% CI: 1.22–1.54; HR: 1.34, 95% CI: 1.03–1.74). Except for age, the effects of modifications from socioeconomic status, lifestyles, and health conditions were non-significant (*p* for interaction <0.05). After multiple imputations for missing data and excluding deaths in the first year of follow-up and participants with a history of the disease and using the Shared-frailty Cox model, sensitivity analysis indicated a robust association between the drinking water source and OA incidence.

**Conclusion:**

Drinking tap water was associated with a low risk of OA among older adults in China. The use of clean water sources as a marker of decreased OA and arthritis risks must be revisited in low- and middle-income countries.

## Introduction

Osteoarthritis (OA) is a chronic pathological joint disease characterized by changes in cartilages and hyperplasia around bones (1). Patients with OA often experience knee joint injury, low activity, poor self-efficacy, severe fatigue, social ability limitation, and great functional disability (2). According to a Global Burden of Disease report, hip and knee OA ranked the 11th highest contributor to global disability and 38th highest in disability-adjusted life years (3). In China, the prevalence rates of mild, moderate, and severe knee OA among 17,459 middle and older people are 1.5, 3.3, and 3.9%, respectively (4), and total and per 100,000 population number of years lived with disability for knee OA are 4,149,628 and 968, respectively (4).

Osteoarthritis pathogenesis is unclear and linked with age, obesity, inflammation, trauma, and genetic and environmental factors. Consumable water in China contains a dangerous concentration of arsenic, fluorine, and sulfates (5), and an excess intake of fluoride and nitrate is associated with an increased risk of OA (6–8). In China, chemical and phosphate fertilizer plants discharge waste gas with fluorine into natural water sources (9, 10). Except for Shanghai and Hainan, the fluorine level for drinking water in most parts of China is over the limit (9, 10). An epidemiological study suggested a connection between OA prevalence and water districts contaminated with perfluorooctanoate (PFOA) and perfluorooctane sulfonate (PFOS) (6). A research using a rat OA model also revealed that (18) F-fluoride is potentially related to early OA changes (11). These empirical results indicate that drinking unprocessed water or polluted water increases the risk of OA possibly through a biological mechanism that fluorides or nitrides exert an adverse influence on the apatite of bone with secondary inflammation and oxidative stress in osteoporosis and osteosclerosis (12).

The global elderly population is at an increased risk of OA (13), and this cohort (aged ≥60 years) is rapidly growing in China (14). Therefore, the association between water contamination and OA incidence in this age group must be examined in the Chinese context. Water source indirectly reflects the quality of drinking water for residents. However, only a few studies were conducted on the association between drinking water sources and OA incidence in older people, especially in low- and middle-income countries.

Considering that OA might be affected by different water sources, this study evaluated the long-term effects of drinking water sources on OA risk among the Chinese elderly population.

## Methods

### Study Design and Subjects

The Chinese Longitudinal Healthy Longevity Survey (CLHLS) is a dynamic longitudinal study of follow-up surveys conducted every 2–4 years that covers 85% of the elderly in 22 provinces in China. From 1998 to 2000, CLHLS recruited participants aged 80 years and above and performed multistage, stratified cluster sampling. Participants aged 65–79 years were additionally recruited since 2002. All the participants were interviewed on determinants of healthy longevity for the elderly, such as socioeconomic characteristics, lifestyle, social interaction, residential environment, cognitive function, and physical/mental condition. This study was approved by the Research Ethics Committee of Peking University (IRB00001052-13074), and written consent was obtained from all participants or their legal representatives.

To date, six waves of surveys have been performed. Individuals still living were followed up in each wave. If a participant has already died when being surveyed, then his/her relatives and/or family members were interviewed for the cause of his/her death, health condition, and utilization of health service before death. The questionnaires were divided into two sections, namely, the “survivors questionnaire” (for participants alive) and the “deceased questionnaire” for the relatives/family members (15). The current study extracted the dataset from 2002, 2005, 2008, 2011, 2014, and 2018 waves. Participants aged 65 years and above were included.

In the first wave (2002), 16,064 individuals were selected. Among which, 12,543 participants with complete information on drinking water sources and OA history at baseline and follow-up surveys were eligible. A total of 599 participants with missing data or ambiguous answers, 2,710 participants with OA at baseline, and 212 participants leaving the place of residence were excluded. A fixed cohort was employed in this study.

### Arthritis Assessment

During the succeeding surveys in the cohort, OA diagnosis was self-reported (“Have you suffered from osteoarthritis?”). The outcome was defined as OA that occurred in the follow-up period for participants without a history of OA at baseline. All interviewees were followed up from baseline until arthritis incidence, dropout, or latest interview.

### Assessment of Water Sources

Water sources were identified by one item: “What source of water source did you mainly drink from at present [well, surface water (river, lake, pond, and pool), spring, and tap water]?”

### Covariates

Several potential confounders were adjusted in the analyses. Sociodemographic data included age, sex, residential place (rural/urban), and education level (illiterate/primary school/high school or above). Lifestyle factors comprised smoking status (never/previously/currently), drinking status (never/previously/currently), dietary diversity scores (low/medium/high), and social activity (yes/no). Health status was composed of body weight (kg), chronic diseases (hypertension, diabetes, heart disease, stroke, pneumonia, and tuberculosis; yes/no), cognitive function [as measured by Mini-Mental State Examination (MMSE) score], and depression status (measured by five items). Detailed definitions of covariates are described in the Appendix methods in Supplementary Material.

### Statistical Analysis

Continuous variables were described as mean ± SD and analyzed by Chi-square test, and categorical variables were described as frequency and proportion (%) and tested by ANOVA or Kruskal–Wallis test. Multivariable Cox regression was used to assess the association between water sources and OA incidence. Drinking well water was set as the reference group. A set of Cox regression models were built: Model 1—adjusting age and gender; Model 2—further adjusting residence, education, income, region, and weight; Model 3—further adjusting social activity and physical activity based on model 2; Model 4—further adjusting drinking alcohol, smoking, and dietary scores; and Model 5—further adjusting chronic disease, depressive symptoms, and cognitive impairment.

Subgroup analyses were conducted to test multiplicative interactions between water sources and covariates by adding a cross-product term in the full multivariable Cox regression model, namely, sex, age, residence, chronic disease, drinking, smoking, or exercise.

Three sensitivity analyses were performed to address the potential influence of dropout. First, deaths in 2005 (second wave) were excluded to ensure that the participants were pre-mortality. Second, individuals with chronic diseases (e.g., hypertension, diabetes, heart attack, stroke, pneumonia, and tuberculosis) were excluded to minimize the hysteretic effects of chronic diseases on the outcome. Third, multiple imputations based on five replications and Monte Carlo simulations were performed to account for missing data because the covariates were disproportional and randomly missing. The Cox model with shared frailty was used to treat the cluster, and participants with equal value were assumed to have shared frailty across different groups in controlled provinces. The effect within the province group was estimated separately to identify the significance of regional heterogeneity.

All analyses were performed using Stata 16.1 (Stata Corporation, College Station, TX, USA). Statistical significance was considered when the two-side *p* was <0.05 (two-sided).

## Results

### Baseline Characteristics of Participants

**Figure 1:**
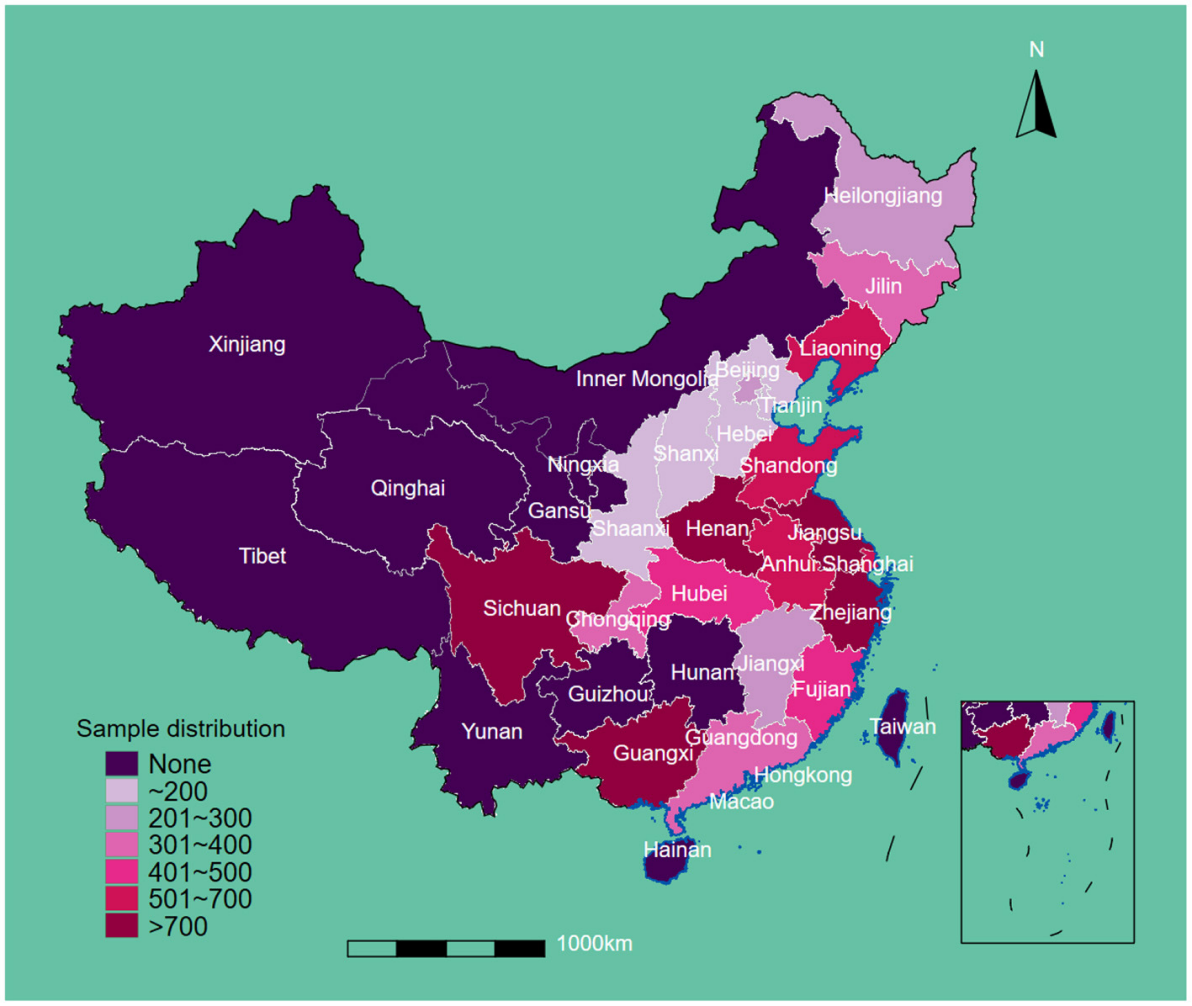
Sample distribution.

This study included 12,543 eligible participants at baseline. The regional sample distribution is displayed in Figure 1. The average age and body weights were 86.71 years and 49.13 kg, respectively. A total of 5,489 (43.8%) men and 7,054 (42.6%) women were enrolled. After the restriction to the longest follow-up, the mean survival time of follow-up was 14.56 years. In the cohort, 1,585 suffered from OA. In terms of drinking water sources, 4,372 people drank from a well, 171 drank from the surface water, 400 drank from the spring water, and 7,600 drank from tap water. More than half of participants were illiterate (7,679, 61.2%), living in rural areas (6,707, 53.5%), never drank alcohol (8,368, 66.9%), never smoked (8,125, 65.0%), and had low leisure time in physical activity (7,099, 56.8%). Most of the participants were absent from regular social activity (10,924, 87.1%), and approximately 40% reported low-level food scores (5,149, 41.1%). Over half of the participants were cognitively impaired (5,951, 56.8%), and the mean score of depressive status was 11.7. In terms of chronic diseases, 1,734 (14.0%) had hypertension, 257 (2.1%) had diabetes, 1,046 (8.4%) had heart attack, 637 (5.1%) had stroke/cardiovascular disease (CVD), 1,425 (11.4%) had pneumonia, and 84 (0.7%) had tuberculosis (Table 1).

**Table 1:** Characteristics of 12,543 participants aged over 65 years and older according to CLHLS.

**Characteristics**	**Total**	**Well**	**Surface**	**Spring**	**Tap water**	***P*-value**
	***N* = 12,543**	***N* = 4,372**	***N* = 171**	***N* = 400**	***N* = 7,600**	
Age (years)	86.7 (11.6)	87.3 (11.9)	87.6 (13.0)	86.8 (12.2)	86.4 (11.4)	<0.001
Weight	49.1 (10.7)	47.5 (10.0)	45.0 (9.6)	44.0 (8.0)	50.5 (11.0)	<0.001
Sex						0.002
Male	5,489 (43.8%)	1,844 (42.2%)	65 (38.0%)	158 (39.5%)	3,422 (45.0%)	
Female	7,054 (56.2%)	2,528 (57.8%)	106 (62.0%)	242 (60.5%)	4,178 (55.0%)	
Years of schooling						<0.001
Illiterate	7,679 (61.2%)	3,074 (70.3%)	123 (71.9%)	288 (72.0%)	4,194 (55.2%)	
Primary school	3,531 (28.2%)	1,082 (24.7%)	37 (21.6%)	91 (22.8%)	2,321 (30.5%)	
Higher school	1,333 (10.6%)	216 (4.9%)	11 (6.4%)	21 (5.3%)	1,085 (14.3%)	
Living area						<0.001
Rural	6,707 (53.5%)	3,463 (79.2%)	128 (74.9%)	313 (78.3%)	2,803 (36.9%)	
City	5,836 (46.5%)	909 (20.8%)	43 (25.1%)	87 (21.8%)	4,797 (63.1%)	
Drinking						<0.001
Never drinking	8,368 (66.9%)	2,785 (63.9%)	118 (69.4%)	287 (71.9%)	5,178 (68.3%)	
Formal drinking	1,575 (12.6%)	532 (12.2%)	21 (12.4%)	29 (7.3%)	993 (13.1%)	
Present drinking	2,562 (20.5%)	1,040 (23.9%)	31 (18.2%)	83 (20.8%)	1,408 (18.6%)	
Smoking						<0.001
Never smoking	8,125 (65.0%)	2,798 (64.2%)	119 (70.0%)	276 (69.2%)	4,932 (65.1%)	
Formal smoking	2,061 (16.5%)	642 (14.7%)	22 (12.9%)	41 (10.3%)	1,356 (17.9%)	
Present smoking	2,323 (18.6%)	921 (21.1%)	29 (17.1%)	82 (20.6%)	1,291 (17.0%)	
Physical activity						<0.001
Low	7,099 (56.8%)	2,863 (65.7%)	128 (75.3%)	311 (78.1%)	3,797 (50.1%)	
Medium	1,425 (11.4%)	403 (9.2%)	15 (8.8%)	20 (5.0%)	987 (13.0%)	
High	3,985 (31.9%)	1,095 (25.1%)	27 (15.9%)	67 (16.8%)	2,796 (36.9%)	
Social activity						<0.001
Never	10,924 (87.1%)	4,036 (92.3%)	162 (94.7%)	369 (92.3%)	6,357 (83.6%)	
Sometimes	1,392 (11.1%)	290 (6.6%)	8 (4.7%)	28 (7.0%)	1,066 (14.0%)	
Always	227 (1.8%)	46 (1.1%)	1 (0.6%)	3 (0.8%)	177 (2.3%)	
Food scores						<0.001
Low	5,149 (41.1%)	1,376 (31.5%)	50 (29.2%)	152 (38.0%)	3,571 (47.0%)	
Medium	3,531 (28.2%)	1,290 (29.5%)	64 (37.4%)	130 (32.5%)	2,047 (26.9%)	
High	3,863 (30.8%)	1,706 (39.0%)	57 (33.3%)	118 (29.5%)	1,982 (26.1%)	
Cognitive impairment						0.12
No	4,528 (43.2%)	1,582 (44.0%)	55 (42.0%)	157 (48.3%)	2,734 (42.5%)	
Yes	5,951 (56.8%)	2,011 (56.0%)	76 (58.0%)	168 (51.7%)	3,696 (57.5%)	
Depression	11.7 (3.3)	12.0 (3.3)	12.7 (3.6)	12.7 (3.1)	11.4 (3.2)	<0.001
Hypertension						<0.001
No	10,669 (86.0%)	3,835 (89.2%)	147 (89.6%)	361 (91.2%)	6,326 (83.9%)	
Yes	1,734 (14.0%)	466 (10.8%)	17 (10.4%)	35 (8.8%)	1,216 (16.1%)	
Diabetes						<0.001
No	12,159 (97.9%)	4,293 (99.3%)	164 (98.2%)	393 (99.2%)	7,309 (97.1%)	
Yes	257 (2.1%)	30 (0.7%)	3 (1.8%)	3 (0.8%)	221 (2.9%)	
Heart attack						<0.001
No	11,376 (91.6%)	4,125 (95.3%)	154 (92.2%)	384 (97.0%)	6,713 (89.1%)	
Yes	1,046 (8.4%)	203 (4.7%)	13 (7.8%)	12 (3.0%)	818 (10.9%)	
Stroke/CVD						<0.001
No	11,825 (94.9%)	4,159 (95.8%)	163 (97.6%)	389 (97.7%)	7,114 (94.2%)	
Yes	637 (5.1%)	182 (4.2%)	4 (2.4%)	9 (2.3%)	442 (5.8%)	
Pneumonia						0.15
No	11,041 (88.6%)	3,865 (89.0%)	150 (89.8%)	363 (91.4%)	6,663 (88.2%)	
Yes	1,425 (11.4%)	480 (11.0%)	17 (10.2%)	34 (8.6%)	894 (11.8%)	
Tuberculosis						0.052
No	12,374 (99.3%)	4,312 (99.6%)	167 (100.0%)	396 (99.5%)	7,499 (99.2%)	
Yes	84 (0.7%)	19 (0.4%)	0 (0.0%)	2 (0.5%)	63 (0.8%)	

*CLHLS, the Chinese Longitudinal Healthy Longevity Survey*.

### Association Between Drinking Water Sources and OA Incidence

As shown in Table 2, the incident rates (per 1,000) of OA are 10.47, 9.36, 11.43, and 7.51. In Model 1, the hazard ratios (HR) (95% CI) for those who drank from well, surface, and spring water were 1.43 (1.30–1.58), 1.25 (0.83–1.88), 1.47 (1.16–1.85), respectively, compared with those drinking tap water. In the multivariable-adjusted Cox regression (Model 5), the associations were attenuated for those drinking well, surface, and spring water but were still significant in the groups drinking from well and spring water (drinking from well: HR for 1.37, 95% CI: 1.22–1.54; drinking from spring water: HR for 1.34, 95% CI:21.03–1.74).

**Table 2:** Hazard ratio (95% CI) for arthritis with drinking source.

**Model**	**Drinking source**			
	**Well**	**Surface**	**Spring**	**Tap water**
Number of deaths	655	23	65	842
Person-years	62,549	2,458	5,685	112,054
Incident rate (per 1,000)	10.472	9.357	11.434	7.514
**HR (95%CI)**				
Model 1	1.43 (1.30–1.58)^*^	1.25 (0.83–1.88)	1.47 (1.16–1.85)^*^	1.00
Model 2	1.32 (1.18–1.47)^*^	1.14 (0.75–1.73)	1.33 (1.04–1.69)^*^	1.00
Model 3	1.33 (1.19–1.48)^*^	1.17(0.78–1.78)	1.36 (1.07–1.73)^*^	1.00
Model 4	1.33 (1.19–1.48)^*^	1.17(0.78–1.77)	1.35 (1.06–1.72)^*^	1.00
Model 5	1.37 (1.22–1.54)^*^	1.07(0.66–1.72)	1.34 (1.03–1.74)^*^	1.00

*Model 1 adjusted for sex age; Model 2 adjusted for sex age, residence, education level, and weight; Model 3 adjusted for sex age, residence, education level, weight, social activity, and physical activity; Model 4 adjusted for sex age, residence, education level, weight, social activity and physical activity, alcohol drinking, and history of smoking; Model 5 adjusted for sex age, residence, education level, weight, social activity and physical activity, alcohol drinking, and history of smoking, chronic disease, depressive status, and MMSE. MMSE, Mini-Mental State Examination*.

* *P < 0.05*.

*The bold values represent reaching a significant threshold*.

### Subgroup Analysis Between Drinking Water Sources and OA Incidence

A series of subgroup analyses was conducted by using pre-defined factors (sex, age, living area, chronic disease, smoking, drinking, and physical activity; Table 3). Compared with tap water, drinking mainly from well and spring water showed a positive association with OA incidence that was generally persistent across subgroups. A significant interaction between drinking water sources and age (*p* < 0.001) was also observed. In terms of drinking mainly from well, a significantly large effect size was observed among female participants, those aged below 80 years, living in rural areas, had no chronic disease, never smoked and drunk, and had no physical activity. In terms of drinking main from spring water, a significantly large effect size was observed among women, those living in rural areas, had chronic diseases or smoking/drinking habits, and participated in physical activity.

**Table 3:** Hazard ratio and 95% CI of OA in participants drinking from different water sources stratified by participants' characteristics.

**Variables**	**Water sources type**				
	**Well**	**Surface**	**Spring**	**Tap water**	***P* for Interaction**
Sex					
Male	1.36 (1.15, 1.62)^*^	0.82 (0.36, 1.83)	1.085 (0.69, 1.72)	1.00	0.42
Female	**1.38 (1.18, 1.61)**	1.28 (0.72, 2.27)	1.62 (1.17, 2.24)^*^	1.00	
Age					
<80	**1.48 (1.28, 1.71)** ^*^	0.88 (0.48, 1.62)	1.37 (0.99, 1.90)	1.00	0.0036^*^
>80	1.19 (0.97, 1.47)	1.55 (0.75, 3.21)	1.43 (0.89, 2.29)	1.00	
Living Area					
Rural	1.44 (1.25, 1.65)^*^	1.09 (0.63, 1.91)	1.39 (1.01, 1.90)^*^	1.00	0.7906
City	1.34 (1.07, 1.68)^*^	1.23 (0.49, 3.12)	1.62 (0.97, 2.70)	1.00	
Disease					
No	1.41 (1.23, 1.62)^*^	0.96 (0.54, 1.73)	1.18 (0.85, 1.63)	1.00	0.1607
Yes	1.28 (1.04, 1.59)^*^	1.32 (0.61, 2.89)	2.00 (1.28, 3.15)^*^	1.00	
Smoking					
Yes	1.37 (1.18, 1.58)^*^	1.23 (0.73, 2.09)	1.58 (1.16, 2.14)^*^	1.00	0.3839
No	1.38(1.14, 1.68) ^*^	0.77 (0.28, 2.11)	1.00 (0.58, 1.73)	1.00	
Drinking					
Yes	1.42 (1.23, 1.63)^*^	1.15 (0.67, 1.96)	1.58 (1.17, 2.12)^*^	1.00	0.3652
No	1.29 (1.05, 1.58)	0.89 (0.33, 2.37)	0.99 (0.56, 1.77)	1.00	
Physical Activity					
Yes	1.27 (1.09, 1.48)^*^	1.05 (0.61, 1.82)	1.38 (1.01, 1.89)^*^	1.00	0.5597
No	1.59 (1.33, 1.90)^*^	1.06 (0.41, 2.69)	1.35 (0.79, 2.34)	1.00	

*MMSE, Mini-Mental State Examination; OA, osteoarthritis*.

* *p < 0.05, model adjusted for sex age, residence, education level, weight, social activity and physical activity, alcohol drinking and history of smoking, chronic disease, depression status, and MMSE*.

*The bold values represent reaching a significant threshold*.

### Sensitivity Analysis

After imputing missing values in the sensitivity analyses of Cox regressions, drinking from the well and spring water was associated with a high risk of OA (HR for well water: 1.31, 95% CI: 1.18–1.46, and HR for spring water: 1.34, 95% CI: 1.05–1.71). Although the individuals who died before the second wave (2005) were excluded, the fitted model was comparable with the models imputing missing values. In addition, the participants drinking from well had a high risk but no significance was observed for those drinking spring water (HR for well water: 1.40, 95% CI: 1.22–1.61). After participants with chronic diseases were excluded, drinking well and spring water was linked with a high risk of OA (HR: 1.38 and 1.31, 95% CI: 1.23–1.55 and 1.02–1.70). Finally, the Cox model with shared frailty showed that drinking well water was positively associated with OA risk, though the cluster effect within provinces was controlled (HR: 1.26, 95% CI: 1.11–1.43; Table 4). Furthermore, drinking well water might be associated with a relatively strong risk of OA in southern (HR: 1.24, 95% CI: 1.01–1.52) and western regions (HR: 1.49, 95% CI: 1.06–2.11), but the *p* for interaction was not supported for regional heterogeneity (*p* > 0.05; Table 5).

**Table 4:** Sensitivity analyses for the association between the water source and OA.

**Water sources type**	**Hazard ratio (95%CI)**			
	**Model 1^a^**	**Model 2^b^**	**Model 3^c^**	**Model 4^d^**
Well	1.40 (1.22–1.61)^*^	1.38 (1.23–1.55)^*^	1.31(1.18–1.46)^*^	1.26(1.11–1.43)^*^
Surface	0.96(0.53–1.75)	1.03(0.64–1.65)	1.16(0.77–1.75)	0.91(0.56–1.49)
Spring	1.12(0.81–1.54)	1.31 (1.02–1.70)^*^	1.34(1.05–1.71)^*^	1.17(0.87–1.56)
Tap water	Ref.	Ref.	Ref.	Ref.

*Models 1, 2, and 3 were adjusted for sex age, residence, education level, weight, social activity and physical activity, alcohol drinking, and history of smoking, chronic disease, depression status, and MMSE. Exclusion of deaths that occurred in the first year of follow-up. Exclusion of participants with a history of the disease. Multiple imputation conducted for missing data. Cox model with shared frailty. MMSE, Mini-Mental State Examination; OA, osteoarthritis*.

* *P < 0.05*.

*The bold values represent reaching a significant threshold*.

a *Exclusion of deaths that occurred in the first year of follow-up*.

b *Exclusion of participants with a history of the disease*.

c *Multiple imputation conducted for missing data*.

d *Cox model with shared frailty*.

**Table 5:** Subgroup analyses for the association between the water source and OA across different regions.

	**Geographical regions**			
**Water source type**	**Northern China**	**Eastern China**	**Southern China**	**Western China**
	**HR (95% CI)**	**HR (95% CI)**	**HR (95% CI)**	**HR (95% CI)**
Well	1.22 (0.89–1.67)	1.20 (0.98–1.48)	1.24 (1.01–1.52)^*^	1.49 (1.06–2.11)^*^
Surface/Spring	2.45 (0.84–7.13)	1.40 (0.90–2.17)	0.98 (0.69–1.39)	1.33 (0.66–2.67)
Tap water	Ref.	Ref.	Ref.	Ref.
*P* for interaction		**0.42**		

*Surface and spring water sources were combined due to limited samples in the specific region. OA, osteoarthritis*.

* *p < 0.05*.

*The bold values represent reaching a significant threshold*.

## Discussion

In this national longitudinal study that includes 12,543 Chinese adults aged 65 years and above, the association between drinking water sources and OA incidence was examined. The primary finding was that drinking tap water was associated with a lower risk of OA compared with drinking well and spring water. For drinking tap water, the HRs of OA were 30% higher for those drinking well water and spring water, regardless of other risk factors. Effect modifications from subgroups were non-significant except for age, indicating that the associations were robust.

To the authors' knowledge, this study was the first to evaluate the association between drinking water sources and OA. Related works have reported about drinking water and arthritis. Acute diarrheal pathogens are often a high-risk issue for residents' unclean water supplies and gastrointestinal disease. Various potential sequelae follow enteric infections, such as diabetes, myocarditis, and reactive arthritis (16). However, the burden of pathogens for health conditions is often temporary. A follow-up survey involving 9,500 residents in Finnish measured the complaints or symptoms of participants after extensive backflow that caused household water contamination (17). The outbreak's long-term consequences were minor in terms of the self-reported symptoms or complaints (17). Therefore, the long-term effect of unprocessed drinking water on health change should be considered in chemicals and minerals. One retrospective cohort study reported that participants who lived in communities where the drinking water from private well water with a high nitrate N content had a great risk of intestinal difficulties and OA (8). Fluoride- and arsenic-contaminated drinking water are positively associated with skeletal fluorosis worldwide, especially in rural areas (18). An increase in groundwater fluoride concentration is a potential risk factor for arthritis in Asian countries (19–21). Approximately 2.58% of the area in Jinghui irrigation district, China groundwater arsenic level exceeded the limit of 10 μg/L for drinking water (22). Although tap water is the best water source, issues about water delivery should be further examined. For example, the WHO *Guidelines for Drinking-water Quality* reported that cadmium contamination of drinking water might occur due to the presence of cadmium as an impurity in the zinc of galvanized pipes or cadmium-containing solders in fittings, water heaters, water coolers, and taps (23). Cadmium can increase bone resorption, affect the activity of osteoclasts and calcium absorption, and impair kidney function, thus leading to osteoporosis, OA, and rheumarthritis (24). The present findings were consistent with the abovementioned evidence. In general, tap water was the most beneficial for decreasing OA risk.

Except for age adjustment, the interactive effect of drinking water sources and OA was robust. Age is a determining risk factor associated with OA (25, 26). The estimated prevalence of OA was 25.03% for lumbar, 21.51% for knee, 20.46% for cervical, and 8.99% for hand in middle-aged and older Chinese adults according to the current meta-analysis (26). Owing to the projected increase in life expectancy, the population of middle-aged and elderly OA patients will also continue to grow. The meta-analysis also revealed a trend of increasing prevalence with age before 70 years old and a slight decrease in the oldest ages (26); this finding was comparable with the present results. This work suggests the need for implementing indoor clean water sources and the development of cost-effective interventions to address the adverse health effects of unprocessed drinking water and reduce the increasing prevalence of OA from the pollutant water use of chemicals and minerals in this vulnerable segment of the population in rural areas.

The underlying biological mechanisms of promoting OA incidence due to long-term intake of unprocessed drinking water remain unclear. Some studies on molecular biology attempted to illustrate the association. Arsenic induces senescence in human articular chondrocytes by increasing the activity of β-galactosidase (SA-β-Gal) and protein expression of p16, p53, and p21 (27). In a rat experiment, increased cartilage senescence and abrasion were found in the rats treated by long-term drinking water with arsenic (0.05 and 0.5 ppm) for 36 weeks compared with those in age-matched control rats (27). The phosphorylation of p38 and JNK and the induction of GATA4-NF-κB signaling and senescence-associated secretory phenotype (SASP) were also enhanced in the rat cartilages (27). This signaling pathway has been used as the most specific explanation for the pathological mechanism of OA (28, 29). For fluoride, in *ex vivo* models and animal studies linked PFOS and PFOA to inflammation (30–32), oxidative stress (33, 34), and disturbance of calcium homeostasis (35). In particular, PFOA increases inflammation by inducing proinflammatory cytokines (32). By binding to peroxisome proliferator-activated receptor (PPAR)—PPAR-α and γ–PFOS, and PFOA trigger changes in bone metabolism that are related to the progression and onset of OA symptoms (6).

This study shows several strengths. First, this work is the first attempt to analyze the effect of drinking water sources on the OA incidence of the Chinese elderly. The findings provide novel evidence for improving the availability of water, sanitation, and hygiene and the prevention of OA in the elderly. Second, this study was based on CLHLS, a large-scale population-based cohort study. Sufficient statistical power was obtained to infer the casual associations of drinking water sources with OA incidence. Finally, three sensitive analyses were conducted to verify the accuracy and credibility of the association, and the results were consistent with those from the analysis of the overall sample.

This study has several limitations. First, sufficient and valid information on the diagnosed OA cases was not available from the dataset. Recall bias could be generated due to retrospective longitudinal design and self-report data. Second, detailed food data were not accessible, and the proxy variable of the dietary pattern cannot be adopted in the baseline questionnaire. Therefore, the association between dietary patterns and drinking water sources cannot be explored. Third, the proportion of participants drinking spring water and surface water was relatively small. A large sample size should be employed to improve the power of statistical inference. Finally, the dose-response relationship of chemical substances and OA (e.g., fluorides or nitrate) cannot be assessed. Further evidence from environmental medicine perspective must be obtained to clarify the underlying mechanism between drinking water sources and OA. The conclusion of this research was proposed carefully.

## Conclusion

Drinking well and spring water was associated with a high risk of OA among the population of age >65 years old in China. Therefore, the association between unprocessed drinking water with a multilevel exposure and OA incidence must be re-examined in low- and middle-income countries. Further longitudinal studies are also needed to confirm the relationship as causal. This work paves the way for an evidence-based intervention for the policymakers to ease the burden of OA related to water pollution for the elderly in such a globally aging population.

## Data Availability Statement

The datasets presented in this study can be found in online repositories. The names of the repository/repositories and accession number(s) can be found below: https://sites.duke.edu/centerforaging/programs/chinese-longitudinal-healthy-longevity-survey-clhls/.

## Ethics Statement

The studies involving human particpants were reviewed and approved by the Institutional Review Board, Duke University (Pro00062871), and the Biomedical Ethics Committee, Peking University (IRB00001052-13074). The patients/participants provided their written informed consent to participate in this study.

## Author Contributions

YZ and TW contributed to the conception, analysis, interpretation of data, drafted the report, and received the final version for publication. YL, YX, SS, JY, XC, XW, and XL contributed to analyzing and interpreting the data, commented on the report, revised the manuscript, and approved the final version for submission. All authors have read and agreed to the published version of the manuscript.

## Funding

This work was supported by the Natural Science Foundation General Project of Chongqing Science and Technology Bureau (Grant No. cstc2020jcyj-msxmX0279) and the Science and Technology Research Project of Chongqing Education Commission (Grant No. KJQN202100432).

## Conflict of Interest

The authors declare that the research was conducted in the absence of any commercial or financial relationships that could be construed as a potential conflict of interest.

## Publisher's Note

All claims expressed in this article are solely those of the authors and do not necessarily represent those of their affiliated organizations, or those of the publisher, the editors and the reviewers. Any product that may be evaluated in this article, or claim that may be made by its manufacturer, is not guaranteed or endorsed by the publisher.

## Abbreviations

CLHLS: China Longitudinal Healthy Longevity Survey
WHO: World Health Organization
OA: osteoarthritis
HR: hazard ratio
CI: confidence interval.
