# Supplementary material for: Association Between Drinking Water Sources and Osteoarthritis Incidence in Chinese Elderly Population: National Population-Based Cohort Study
Source: Front Med (Lausanne). 2022 Feb 3;8:759514. doi: 10.3389/fmed.2021.759514 (PMC8851347; doi:10.3389/fmed.2021.759514)
Supplement: Supplementary file 1 [file Data_Sheet_1.docx]

***Appendix Methods.***

**Detailed definitions of covariates in our study.**

Age (years) was calculated based on the difference between the follow-up year and Georgian calendar dates. The current residence was dichotomized as “Rural area” and “City area.” Education level was divided based on years of schooling (0 years, 1-6 years, and >6 years). Smoking and drinking status was defined as “Never,” “Formal,” and “Current.” Dietary diversity scores were calculated according to eight food groups (staple food, fresh fruit, vegetable, meat, fish, sugar). Types of staple food included rice, corn, wheat, half rice, and half wheat. The amount of staple food was grouped by quintile and meant scores from 1 to 5. In addition, a score of 0 indicated that the individual did not take grain as a staple food. The frequency of fruit and vegetable intake was coded as “every day or almost every,” “quite often,” “occasionally,” and “rarely or never.” The scores ranged from 1 to 4, with higher points reflecting higher times for intake. The rest of the five food were recoded as “almost every day,” “least once per week,” “least once per month,” “occasionally,” and “never.” The scores for these food groups ranged from 1 to 5, with higher scores representing higher frequency for intake. Therefore, dietary diversity scores were summed for all eight foods ranging from 6 to 24, with higher scores equating to abundant dietary diversity (Wang et al. 2020). Weight was calculated in kilograms via a weighting scale. Social activity was defined as whether one took part in social activity, and the item was the score at 1 “never,” 2 “sometimes,” 3 “always.” The Chinese version of the Mini-Mental State Examination (MMSE) evaluated the global cognitive function. MMSE has four dimensions of cognitive orientation, calculation, recall, and language capacity, with a total of 24 items scoring from 0 to 30, and the higher scores indicate a higher level of dependence for the respondents (Zeng et al. 2010). The elderly who obtained 24 scores and above were defined as “normal cognitive function,” while those who scored less than 24 were evaluated to be “cognitive impairment” (Lei et al. 2020). Six common geriatirc diseases were considered adjusted variables: hypertension, diabetes, heart disease, stroke, pneumonia, and tuberculosis. Depressive symptoms were measured using 5 items as follows: 1. Do you always look on the bright side of things? 2. Are you as happy as when you were young? 3. Do you often feel fearful or anxious? 4. Do you feel the older you get, the more useless you are? And 5. I can make my own decisions concerning my personal affairs. The 5-point response scale to each item ranged from “never” to “always.” Depressive symptoms were the sum of 5 items. Geographical regions were divided into four sub-groups based on social context, geographical features and economic development (Yao et al. 2020): Northern China (Beijing, Tianjing, Heibei, Shanxi, Shandong, Liaoning, Jilin, and Heilongjiang Provinces), Eastern China (Shanghai, Anhui, Shanxi, Zhejiang, Fujian, and Jiangsu provinces), Southern China (Guangdong, Guangxi, Henan, Hainan, Hubei, and Hunan provinces), and Western China (Sichuan and Chongqing provinces).

**Reference**

Wang Z, Pang Y, Liu J, Wang J, Xie Z, Huang T. Association of healthy lifestyle with cognitive function among Chinese older adults. Eur J Clin Nutr. 2020 Oct 28.

Yi Z, Vaupel JW. Functional capacity and self–evaluation of health and life of oldest old in China. J Soc Issues. 2010;58(4):733–48.

Lei X, Bai C. Cognitive function and mental health of elderly people in China: findings from 2018 CLHLS survey. China Popul Dev Stud. 2020;3:343–51.

Yao Y, Cao K, Zhang K, et al. Residential Proximity to Major Roadways and Prevalent Hypertension Among Older Women and Men: Results From the Chinese Longitudinal Healthy Longevity Survey. Front Cardiovasc Med. 2020;7:587222. Published 2020 Nov 17. doi:10.3389/fcvm.2020.587222
